# Clinical factors affecting salivary transferrin level, a marker of blood contamination in salivary analysis

**DOI:** 10.1186/s12903-018-0510-x

**Published:** 2018-03-21

**Authors:** Jeong-Hyun Kang, Yeon-Hee Lee, Hong-Seop Kho

**Affiliations:** 1 Department of Oral Medicine and Oral Diagnosis, School of Dentistry and Dental Research Institute, Seoul National University, 101 Daehak-ro, Jongno-gu, Seoul, 03080 South Korea; 2 Department of Orofacial Pain and Medicine, Kyung Hee University Dental Hospital, 613 Hoegi-dong, Dongdaemun-gu, Seoul, 130-701 South Korea; 3 Institute on Aging, Seoul National University, 1 Gwanak-Ro, Gwanak-Gu, Seoul, 08826 South Korea

**Keywords:** Saliva, Blood, Transferrin, Gingival inflammation

## Abstract

**Background:**

Diagnostic value of whole saliva may be compromised when blood contamination is present in saliva samples. Measuring transferrin level in saliva samples has been used for detecting the level of blood contamination in saliva. The aim of this study was to investigate the validity of transferrin as a proper biomarker for blood contamination in whole saliva.

**Methods:**

Thirty younger (mean age: 25.9 ± 2.1 years) and twenty older (mean age: 65.1 ± 9.0 years) females were included. The index reflecting overall gingival inflammation (total gingival index), salivary flow rate, and salivary concentration and secretion rate of transferrin of each subject were analyzed.

**Results:**

Salivary transferrin concentrations and secretion rates were higher in the younger females than in the older ones despite a lower total gingival index in the younger females. The total gingival index showed no significant correlations with the concentration or secretion rate of transferrin in either unstimulated or stimulated whole saliva of younger and older subjects. The salivary concentration of transferrin showed negative correlations with the flow rate of saliva in both the younger and older groups. There were significant positive correlations between the salivary concentrations and secretion rates of transferrin in both the younger and older groups.

**Conclusions:**

Salivary transferrin levels could be affected by other factors as well as the level of blood contamination. The influences of age, gonadal hormones, salivary flow rate, and chewing performance need to be considered when using the salivary level of transferrin as a blood contamination marker.

## Background

The diagnostic value of saliva has been focused due to the advantages of its simple and non-invasive collection procedures. For salivary diagnostics, whole saliva rather than glandular saliva has been usually used, and whole saliva is a mixture of exocrine secretions of the salivary glands, microbes, mucosal exudates, gingival crevicular fluid, and desquamated oral epithelial cells. Therefore, the diagnostic value of whole saliva may be compromised when blood contamination, originating either from the loss of mucosal integrity or from gingival exudates, is present. Because the analyte levels are much higher in blood compared to those in saliva, when blood leakage reaches a certain level, the salivary concentrations of analytes are abnormally elevated [1, 2].

Several methods have been proposed to determine blood contamination levels in whole saliva. The first method is to detect a blood tint in a saliva sample by the naked eye [3]. Second, strips for urinalysis have been used [4]. Both methods have relatively low levels of sensitivity [1], and the second method has a possibility of false-positive [5]. The other methods involve measuring the salivary levels of plasma proteins such as hemoglobin [6], albumin [7], and transferrin [1, 2, 8]. Among these methods, measuring the transferrin levels in saliva samples has been regarded as the most reliable method for detecting the level of blood contamination in saliva. However, recent research findings have suggested that this method may also have drawbacks in clinical and research applications [9–13]. Thus, the aim of study was to investigate whether salivary transferrin level could be a proper marker for the level of blood contamination in whole saliva samples.

## Methods

### Participants

To investigate the factors that could influence salivary levels of transferrin, we re-analyzed the data from our two previous papers [10, 14]. One study explored changes in salivary hormones throughout the menstrual cycle in 30 younger healthy females (mean age: 25.9 ± 2.1 years) [10]. The other investigated salivary cytokine levels in patients with a specific oral mucosal pain condition [14]. This study included 20 healthy age-matched females as a control group (mean age: 65.1 ± 9.0 years). We re-analyzed the data from the 30 younger women from the first study and the 20 older women (the control group) from the second study. For younger females, the data obtained in their follicular phases, when oral examinations including measuring the gingival index were conducted, were used in the present study.

### Clinical evaluation

In both studies, the subjects received oral examinations and were confirmed to have intact oral mucosal integrity and no oral mucosal diseases. The extent of gingival inflammation was evaluated by the total gingival index, a modified version of Löe’s gingival index, which reflects sum of gingival inflammation of all remained teeth. To obtain the total gingival index, four gingival areas (facial, mesial, distal, and lingual) adjacent to each tooth were assessed and were given a score from 0 to 3. The scores from the four areas of each tooth were totaled and divided by four to give an average score for each tooth. By adding the average scores from all teeth, the total gingival index for each individual was obtained.

### Collection of whole saliva samples

In both studies, the same methods were used to collect saliva samples. Saliva samples were collected in the morning to reduce circadian variations in salivary compositions. Participants were instructed to abstain from eating and drinking for about two hours prior to the collection of samples. Unstimulated whole saliva (UWS) was collected for 10 min by the spitting method. Stimulated whole saliva (SWS) was collected for 5 min with the chewing of 1 g of gum base. The flow rates were expressed as mL/min.

### Determination of blood contamination in saliva samples

In both studies, the transferrin levels in saliva samples were determined by the enzyme immunoassay method using the same commercial kits (Salimetrics, State College, PA, USA). Secretion rates of transferrin were calculated by multiplying the salivary flow rates and their corresponding concentrations of transferrin.

### Statistics

Based on the Kolmogorov-Smirnov normality test, the data were normally distributed; therefore, parametric tests were applied. The Student’s t-test and Pearson’s correlation analysis with Bonferroni’s correction were used. *P*-values less than 0.05 were considered statistically significant.

## Results

### Age, oral conditions, salivary flow rate, and concentration and secretion rate of transferrin

The total gingival indices which reflect the degree of overall gingival inflammation were significantly lower (*P* <  0.001) and the tooth number was significantly higher (*P* = 0.003) in the younger females than in the older ones. However, transferrin concentrations were higher in the younger females than in the older ones, although there was no statistically significant difference. Because of their higher salivary flow rates, the secretion rates of transferrin were significantly higher in the younger females in both UWS (*P* <  0.001) and SWS (*P* < 0.001) (Table 1).

**Table 1:** Age, oral conditions, salivary flow rate, and concentration and secretion rate of transferrin in younger and older female subjects

		Younger (*n* = 30)	Older (*n* = 20)	*P* value
Age (years)		25.9 ± 2.1	65.1 ± 9.0	< 0.001^**^
Tooth number		28.0 ± 2.0	24.9 ± 6.0	0.003^**^
Total gingival index		17.7 ± 16.0	23.2 ± 10.6	< 0.001^**^
Flow rate (mL/min)	UWS	0.46 ± 0.27	0.20 ± 0.16	0.078
	SWS	1.54 ± 0.48	0.83 ± 0.43	0.556
Concentration of transferrin (mg/dL)	UWS	1.16 ± 0.96	0.65 ± 0.82	0.357
	SWS	0.49 ± 0.38	0.29 ± 0.30	0.227
Secretion rate of transferrin (μg/min)	UWS	4.18 ± 2.87	0.90 ± 0.84	< 0.001^**^
	SWS	6.62 ± 4.44	1.62 ± 0.90	< 0.001^**^

UWS, unstimulated whole saliva; SWS, stimulated whole saliva

Total gingival index reflects the degree of overall gingival inflammation in the oral cavity

Secretion rates of transferrin were calculated by multiplying the flow rates of saliva and their corresponding concentrations of transferrin

The results in the table are from our two previous studies. The data from younger females were adapted from Lee et al. [10] and those from older females were adapted from Suh et al. [14]

^**^P < 0.01 by the Student’s t-test

### Correlations among total gingival index, salivary flow rate, and concentration and secretion rate of transferrin in UWS and SWS

The total gingival index had no significant positive correlation with either the concentration or secretion rate of transferrin in both UWS and SWS of younger and older female subjects (Table 2). However, the correlation coefficient was 0.462 (*P* = 0.010) between the total gingival index and the concentration of transferrin in the SWS from the younger females (Table 2). The salivary concentration of transferrin showed negative correlations with the flow rate of saliva in both the younger and older groups, but significant differences were found only in SWS in both younger (*r* = − 0.506, *P* = 0.004) and older (*r* = − 0.644, *P* = 0.004) participants. There were significant positive correlations between the salivary concentrations and secretion rates of transferrin for both UWS and SWS of younger and older groups (*P* < 0.001) (Table 2).

**Table 2:** Correlations among total gingival index, salivary flow rate, and concentrations and secretion rates of transferrin in UWS and SWS in younger and older females

	Flow rate	Concentration of transferrin	Secretion rate of transferrin	Flow rate	Concentration of transferrin	Secretion rate of transferrin
In UWS of younger females				In SWS of younger females		
Total gingival index	−0.246	0.109	−0.122	− 0.341	0.462	0.325
Flow rate		−0.455	0.119		−0.506^*^	−0.110
Concentration of transferrin			0.666^**^			0.879^**^
In UWS of older females				In SWS of older females		
Total gingival index	0.004	0.109	0.283	0.209	0.059	0.041
Flow rate		−0.352	0.062		−0.644^*^	−0.360
Concentration of transferrin			0.847^**^			0.809^**^

UWS, unstimulated whole saliva; SWS, stimulated whole saliva

Total gingival index reflects the degree of overall gingival inflammation in the oral cavity

Secretion rates of transferrin were calculated by multiplying the flow rates of saliva and their corresponding concentrations of transferrin

The results in the table are from our previous studies [10, 14]

^*^P < 0.0083, ^**^P < 0.0017 by the Pearson’s correlation analysis with Bonferroni’s correction

## Discussion

From the results of the present study, several factors which could influence the level of transferrin in whole saliva could be derived. Because the subjects had intact mucosal integrity, gingival inflammation might be the only source of blood contamination in saliva samples. The total gingival indices were lower in the younger females than in the older ones, but transferrin concentrations were higher in the younger females than in the older ones. There are two possible reasons for these results. Primarily, younger individuals may exhibit better transferrin synthesis compared to older ones. One previous report mentioned an age-related decrease in salivary transferrin levels [13], which supports our results. Second, the levels of gonadal hormones and the state of menopause might affect the synthesis of transferrin, an iron-containing protein. In the proliferative phase such as the follicular and ovulatory phases, the level of transferrin in blood needs to be elevated to cope with the increased demand of the active metabolism of proliferating endometrial cells [12]. Thus, post-menopausal women showed decreased transferrin levels in blood compared to pre-menopausal women [9]. Interestingly, our previous study showed that the salivary concentration of transferrin during the ovulatory phase was higher than those during other phases, although there were no statistically significant differences [10]. Therefore, the influences of age and gonadal hormones could explain the higher concentrations and secretion rates of salivary transferrin in the younger females compared to the older ones despite the lesser degree of gingival inflammation in the younger subjects. Additionally, one animal study proposed endogenous transferrin synthesis in parotid acinar cells and active transportation of transferrin to parotid acinar cells after its synthesis in hepatic cells [11], suggesting that transferrin in whole saliva may not be solely a product of blood.

We found that the concentrations of transferrin in UWS were higher than those in SWS. The salivary concentrations of transferrin also had negative correlations with the flow rates of saliva. These results indicate that salivary flow rate affects the level of transferrin in saliva and that dilution effects occur in the stimulated condition.

The total gingival index showed no positive correlation with transferrin concentration in either UWS or SWS, but the correlation coefficient between the total gingival index and the concentration of transferrin was close to the statistical significant level only in the SWS of the younger females. The younger females may have had better chewing performance during the collection of SWS, because the younger females had more teeth than the older ones and usually younger adults had a greater biting force than older ones. Therefore, the possibility of the leakage of gingival exudates into saliva during chewing could be higher in the younger females as compared to the older ones. Tooth mobility during chewing could give rise to increased blood vessel transudation, which could be related to increases in exudates from the gingival crevice, and could ultimately increase the level of blood contamination in saliva [15].

## Conclusions

Diagnostic studies using saliva usually include quantitative analyses of whole saliva samples, and the reliability and validity of salivary data are affected by the level of blood contamination in these saliva samples. When using the salivary level of transferrin as a blood contamination marker, the influences of age, gonadal hormones, salivary flow rate, and chewing performance need to be considered. Further studies are also needed to identify better markers for detecting blood contamination in saliva samples.

## Abbreviations

SWS: Stimulated whole saliva
UWS: Unstimulated whole saliva
